# Commentary regarding “on-orbit sleep problems of astronauts and countermeasures”

**DOI:** 10.1186/s40779-018-0185-2

**Published:** 2018-10-30

**Authors:** Joseph John Bevelacqua, Seyed Mohammad Javad Mortazavi

**Affiliations:** 1 Bevelacqua Resources, 343 Adair Drive, Richland, WA99352 USA; 2 Department of Electrical Engineering, Biophotonics Lab, University of Wisconsin Milwaukee, 3200 N Cramer St, Milwaukee, WI53211 USA; 3 Doss Lab (R-432), Fox Chase Cancer Center, 333 Cottman Avenue, Philadelphia, PA19111 USA

**Keywords:** Space radiation, Astronauts, Sleep problems, Light flashes, Phosphenes

## Abstract

This commentary addresses the article by Wu et al. entitled “On-orbit sleep problems of astronauts and countermeasures”. In this article, the authors discussed the sleep problems of astronauts. Despite its challenging topic, the paper authored by Wu et al. has at least one major shortcoming. This issue is related to the observation that the sleep pattern of astronauts can be disturbed by light flash phenomenon. Since the first report by astronaut E.E. Aldrin in 1969, many astronauts have reported light flashes. These visually perceived flashes of light occurred in different shapes but they apparently moved across the visual field of astronauts and possibly caused, at least to some extent, sleep problems. Moreover, the countermeasures proposed by the authors may improve astronauts’ sleep pattern, but they do not address the root cause of the light flashes (i.e., heavy ion interactions outside the shielding provided by the Earth’s magnetosphere). A possible approach to reducing light flashes is available using the fact that much of the galactic cosmic radiation (GCR) spectrum is composed of ions that can be diverted from the spacecraft using electromagnetic fields. Possible design parameters and the requisite electric and magnetic field strengths to successfully deflect GCR radiation are outlined.

## Background

The findings of a recent National Aeronautics and Space Administration (NASA)-funded study published in the Lancet Neurology journal showed sleep deficiency in astronauts not only during space shuttle and International Space Station (ISS) missions, but also from the start of a three-month preflight training interval [1]. Wu et al. recently published an article entitled “On-orbit sleep problems of astronauts and countermeasures” have addressed the issue of sleep in space as a new medical research frontier [2]. In their review, the authors discussed the sleep problems of astronauts. Wu et al. also introduced different factors that may cause sleep problems in space. Moreover, Wu et al. highlighted the effects of sleep problems in astronauts on their mental and physical health. Finally, Wu et al. have proposed different countermeasures for sleep disturbance in spaceflight.

## The issue of light flashes

**Fig. 1:**
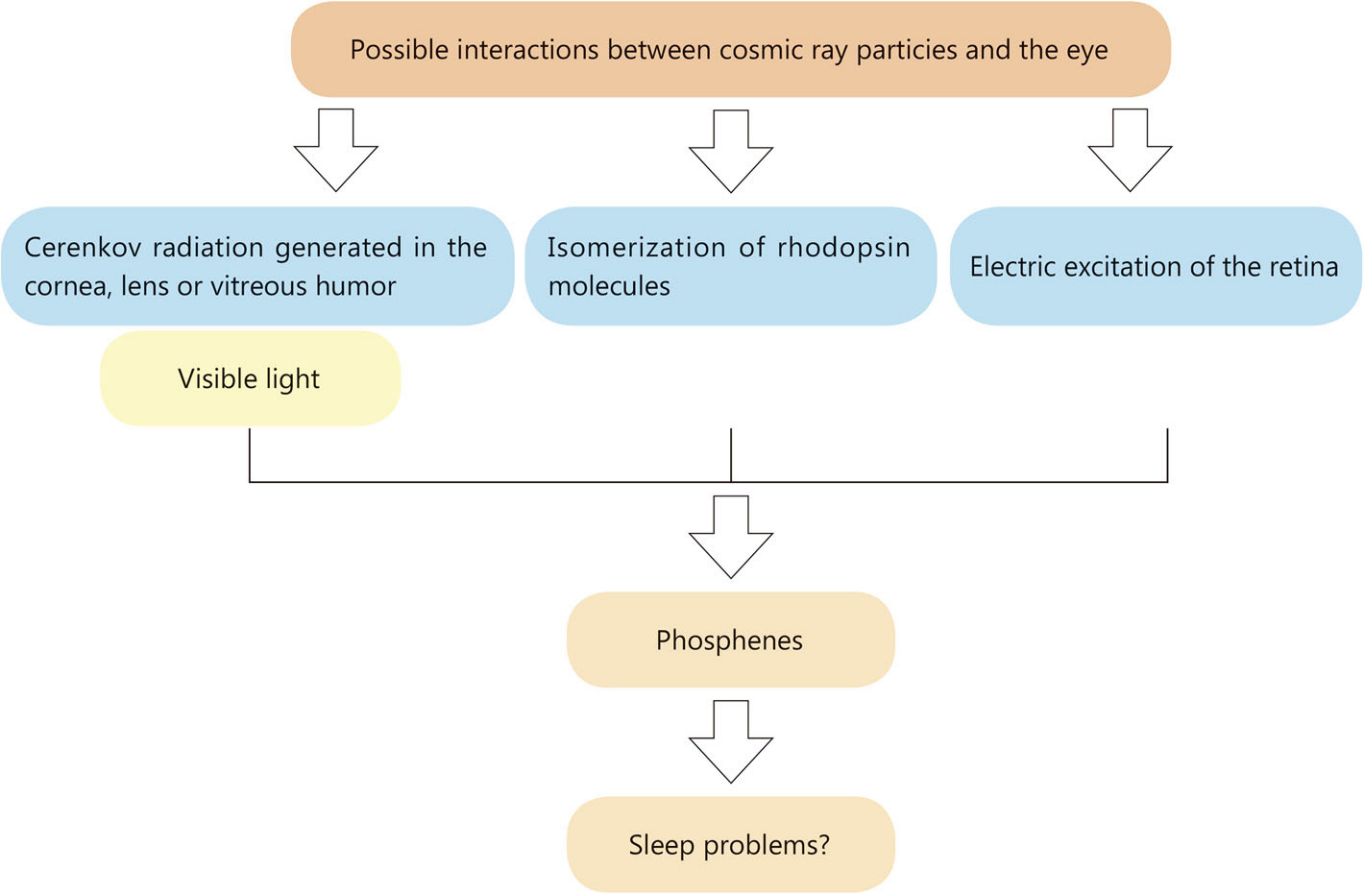
The factors possibly involved in generating visually perceived flashes of light (phosphenes)

Some evidence shows that, at least to some extent, the light flashes seen by astronauts are linked to charged particles traversing the retina. Although the paper authored by Wu et al. is a well-structured informative article, it has at least one major shortcoming. The main shortcoming of this paper comes from this point that the authors ignored the potential issue of light flashes. These flashes of light are produced by two primary mechanisms initiated by galactic cosmic radiation incident on the spacecraft that interacts with the eye. Since the first report by astronaut E.E. Aldrin, one of the first two humans to land on the Moon on July 21, 1969, the majority of astronauts on Apollo, Skylab, and MIR have reported light flashes. Although these flashes of light occurred in different shapes, they apparently moved across the visual field of astronauts [3]. Indirect Cerenkov light generated by nuclear interactions and radioactive decay in the bulk of the eye and direct excitation of the nerve fibres in the back of the eye and/or radical excess near the retina, are the two mechanisms proposed for generation of light flashes in astronauts’ eyes [4]. Figure 1 shows the factors possibly involved in generating phosphenes including visible light due to Cerenkov radiation emitted in different eye sections such as cornea, lens or vitreous humor, as well as the isomerization of rhodopsin molecules and electric excitation of the retina are proposed causes [3].

## Light flashes, circadian rhythm and sleep problems

As reported by Fuglesang et al. [5], light flashes are linked to astronauts’ sleep problems. According to their report, while 70% of astronauts observed light flashes before going to sleep in a dark environment, 12 astronauts reportedly could not fall asleep and even one astronaut occasionally woke up because of light flashes [5]. A more recent report claims that about 80% of people who travel in space have experienced sudden light flashes [6]. Although the issue of light flashes is of importance in sleep problems during space mission [3], previous studies on astronauts’ sleep problems had not pay attention to this issue. Further research on different aspects of this issue, especially during longer space journeys such as Mars missions, is needed. For example, it is not still clear whether light flashes can disrupt the circadian rhythm. It is worth noting that Cherenkov light and scintillation in the eye lens are among several explanations suggested for light flashes [6]. Therefore, it can be hypothesized that these light flashes are capable of disrupting the Circadian rhythm.

Researchers have used irradiation with ^12^C ions to stimulate radical production, propagation and recombination leading to photoluminescence. This study showed that exposure to ^12^C bleached retinal rod outer segment (RdOS) rhodopsin. However, these researchers also observed increased structural damage with radiation dose. They concluded that recombination of radicals could be responsible for the generation of photons with subsequent bleaching of rhodopsin [7].

In this light, disrupted Circadian rhythm or visually induced arousal frequency due to flashes of light generated by possible interaction between cosmic ray particles and the eye can be among the potential causes of sleep problems in astronauts. The quantification of the light flash was addressed by Fuglesang et al. [8]. These researchers indicate that about 50% of the astronauts noted the flashes were too irregular to determine their frequency. Only 20% of the astronauts suggested that the light flashes sometimes disturbed their sleep. Given the critical nature of space travel having 20% of the crew in a sleep deprived state could have a critical impact on mission success. Therefore, light flashes are a potential cause of sleep disturbance in spaceflight. Quantifying these events as a “key issue” is debatable, but the impairment of 20% of the crew justifies a measure of concern which is the point of this commentary.

The specific mechanism affecting sleep disturbance is open to question and merits further investigation. In addition to a shift in Circadian rhythm noted previously, the mechanism could be an increase in visually induced arousal frequency. The exact mechanism should be determined by a comprehensive research program to enhance the health and safety of astronauts.

### Countermeasures for astronauts’ sleep problem

Medication for sleep problems are frequently used by astronauts [9, 10]. Wu et al. also “proposed seven kinds of countermeasures for sleep disturbance in spaceflight, including pharmacologic interventions, light treatment, crew selection and training, and Traditional Chinese Medicine”. Although these countermeasures may improve an astronaut’s sleep pattern, they do not address the root cause of the light flashes (i.e., the cosmic radiation present in the space radiation environment (Fig. 1)) [11].

The countermeasures suggested by Wu et al. [2] do not reduce the cosmic ray induced eye interactions. During a deep space mission, astronauts are exposed to galactic cosmic radiation (GCR) as well as solar particle events (SPEs) [12–15]. Attenuation of GCR is difficult because the constituent radiation types are varied and the spectrum of energies is broad. Accordingly, GCR is difficult to shield [16, 17]. Bulk shielding is heavy and launch weight restrictions limit the amount of material that can be incorporated into the spacecraft shell or shielded enclosures. A possible approach to reducing light flashes is available using the fact that much of the GCR spectrum is composed of ions that can be diverted from the spacecraft using electromagnetic fields. Possible design parameters and the requisite electric and magnetic field strengths to successfully deflect GCR radiation were previously discussed [16].

## The key issue of artificially-generated magnetic fields

NASA believes that space crew health and performance is critical to successful deep space manned explorations. The Human Research Program (HRP) explores and mitigates the significant risks to human health and performance and provides basic countermeasures and technologies for manned space journeys [18]. Major hazards such as radiation, microgravity, and hostile environments are among the risks which should clearly be addressed. Risk of acute radiation syndromes due to solar particle events (SPEs), risk of cardiovascular disease and other degenerative tissue effects from radiation exposure and secondary spaceflight stressors, and risk of radiation carcinogenesis have particularly been addressed. Artificially-generated magnetic fields seems to be a key area of research and technology development that enables deep space manned spaceflights. Artificially-generated magnetic fields can also be considered as a potential countermeasure to sleep disruptions caused by space radiation. Researchers at Florida Institute of Technology have recently proposed a novel architecture for a radiation-protected deep space expedition [19]. To provide artificially-generated magnetic fields, a number of relatively small independent mobile satellites containing superconducting coils which reduce the radiation flux passing through the volume occupied by the spaceship, have been used to form a protective array around the spaceship. As magnetic fields do not change GCR and SPE particle energy, astronauts’ radiation dose inside the shielded region is reduced by the same proportion as the number density of the particles [19].

The electromagnetic deflector concept has the potential to reduce the GCR impacting the eye that would alleviate the interactions that create the light flashes. Incorporation of an electromagnetic deflector into a spacecraft design would provide relief from the light flashes that impact an astronaut’s sleep pattern. The use of the deflector in conjunction with the countermeasures proposed by Wu et al. would significantly improve an astronaut’s work environment. However, potential limitations of artificially-generated magnetic fields such as the cost and complexity as well as insufficiency at higher energies should be fully evaluated. A recent simulation study indicated that the magnetic fields generated by the magnet were sufficient to deflect incoming particles away from the simulated capsule at low energies (< 50 MeV). However, they only halved the flux at higher energies (> 100 MeV) [20]. Regarding the cost, since the largest NASA missions may cost more than a billion dollars, the high cost of effective radiation shielding can be justified.

### Other options for artificially-generated electromagnetic fields

The discussion of Bevelacqua [16] provided conventional design strategies for field generation. There are other options that may merit future consideration for spacecraft applications. An interesting option includes magnetic armor to mitigate the effects of SPE and GCR radiation.

Chesney proposed a novel swarm-bot technology (SBT) approach for protecting astronauts from ionizing radiation during a deep space mission [21]. Its design focuses on the Orion spacecraft with command module, astronaut habitat, and service module, but does not alter these structures or their configuration. SBT proposes to deploy a number of relatively small independent mobile satellites, each containing superconducting coils that form a protective array around the spaceship. These satellites will generate diverging or converging magnetic lenses. The resulting magnetic fields would be designed to reduce the radiation flux within the spatial volume occupied by the spaceship. The SBT is designed to be capable of altering the configuration of the satellite array. As proposed, the individual units could be moved either by their own propulsion or by a designated “tug boat” to form an appropriate array to protect astronauts against changing radiological conditions.

Given their essentially isotropic generation within the universe, SBT protection against GCRs requires a spherically symmetric magnetic shield which could be created by a regular polyhedron array of magnets. SPEs are generated by Solar eruptions and are confined to a more limited solid angle. Upon detection of a SPE, the SBT array would need to be configured to form a network that was oriented in the direction of the incoming radiation. The capability of the SBT to alter its geometric arrangement in a timely manner is an advantage over the fixed “magnetic armor” concept, but it must be designed to accommodate bounding radiological conditions. This is important because SPEs can include short-lived events with intense radiological conditions. The SBT technology would function to improve the safety of astronauts when encountering elevated ionizing radiation fields. Improving astronaut health and safety is a goal of NASA’s Human Research Program.

### NASA human research program

The long-term performance of a spacecraft’s crew will depend on their health and safety. Ensuring the crew’s safety is an essential element for the successful human exploration beyond low earth orbit.

NASA’s Human Research Program (HRP) [22–24] is designed to investigate and mitigate the highest risks to human health and performance. These risks are mitigated by providing essential countermeasures and technologies for human space exploration. As outlined by NASA these risks include “physiological and performance effects from hazards such as radiation, altered gravity, and hostile environments, as well as unique challenges in medical support, human factors, and behavioral health support”. The HRP utilizes an Integrated Research Plan (IRP) [23] to identify the approach and research activities to address the risks identified in NASA report [22]. These research activities are assigned to specific elements within the program. The Human Research Roadmap [24] is the web-based tool that communicates the IRP content, evidence reports, external reviews of HRP research, and general HRP organizational information.

## Conclusions

Astronaut safety is a key priority for sustained spaceflight. Sleep interruption can negatively affect mission success and merits additional study. The light flash phenomenon needs to be better understood as well as its biological impact such as interruption of Circadian rhythm or visually induced arousal frequency. When combining the countermeasures of Wu et al. with an electromagnetic deflector, astronaut safety has the potential to be enhanced by improving sleep quality.

## Abbreviations

GCR: Galactic cosmic radiation
HRP: Human Research Program
IRP: Integrated Research Plan
ISS: International Space Station
NASA: National Aeronautics and Space Administration
RdOS: Rod outer segment
SPE: Solar particle events
